# Novel Blended Learning on Artificial Intelligence for Medical Students: Qualitative Interview Study

**DOI:** 10.2196/65220

**Published:** 2025-05-26

**Authors:** Zoe S Oftring, Kim Deutsch, Daniel Tolks, Florian Jungmann, Sebastian Kuhn

**Affiliations:** 1 Institute for Digital Medicine, Philipps University Marburg and University Clinic Giessen & Marburg, Baldingerstrasse 1, Marburg, 35042, Germany, 49 (0)6421 ext 58; 2 Department of Paediatrics, University Clinic Giessen & Marburg, Marburg, Germany; 3 Institute of Educational Science, Johannes Gutenberg University, Mainz, Germany; 4 Institute of Anatomy, Rostock University Medical Centre, Rostock, Germany; 5 Professorship in Health Management, International University of Applied Science, Hamburg, Germany; 6 Xcare Group Radiology, Nuclear Medicine and Radiotherapy, Saarlouis, Germany

**Keywords:** digital transformation, artificial intelligence, clinical AI, chatbot, digital literacy, medical education, medical students, medical curriculum, qualitative content analysis, medical studies

## Abstract

**Background:**

Artificial intelligence (AI) systems are becoming increasingly relevant in everyday clinical practice, with Food and Drug Administration–approved AI solutions now available in many specialties. This development has far-reaching implications for doctors and the future medical profession, highlighting the need for both practicing physicians and medical students to acquire the knowledge, skills, and attitudes necessary to effectively use and evaluate these technologies. Currently, however, there is limited experience with AI-focused curricular training and continuing education.

**Objective:**

This paper first introduces a novel blended learning curriculum including one module on AI for medical students in Germany. Second, this paper presents findings from a qualitative postcourse evaluation of students’ knowledge and attitudes toward AI and their overall perception of the course.

**Methods:**

Clinical-year medical students can attend a 5-day elective course called “Medicine in the Digital Age,” which includes one dedicated AI module alongside 4 others on digital doctor-patient communication; digital health applications and smart devices; telemedicine; and virtual/augmented reality and robotics. After course completion, participants were interviewed in semistructured small group interviews. The interview guide was developed deductively from existing evidence and research questions compiled by our group. A subset of interview questions focused on students’ knowledge, skills, and attitudes regarding medical AI, and their overall course assessment. Responses were analyzed using Mayring’s qualitative content analysis. This paper reports on the subset of students’ statements about their perception and attitudes toward AI and the elective’s general evaluation.

**Results:**

We conducted a total of 18 group interviews, in which all 35 (100%) participants (female=11, male=24) from 3 consecutive course runs participated. This produced a total of 214 statements on AI, which were assigned to the 3 main categories “Areas of Application,” “Future Work,” and “Critical Reflection.” The findings indicate that students have a nuanced and differentiated understanding of AI. Additionally, 610 statements concerned the elective’s overall assessment, demonstrating great learning benefits and high levels of acceptance of the teaching concept. All 35 students would recommend the elective to peers.

**Conclusions:**

The evaluation demonstrated that the AI module effectively generates competences regarding AI technology, fosters a critical perspective, and prepares medical students to engage with the technology in a differentiated manner. The curriculum is feasible, beneficial, and highly accepted among students, suggesting it could serve as a teaching model for other medical institutions. Given the growing number and impact of medical AI applications, there is a pressing need for more AI-focused curricula and further research on their educational impact.

## Introduction

### Background

The digital transformation in the health care system represents a fundamental process of change and innovation that is altering the roles, competencies, and cooperation of doctors to a large extent [1]. Eric Topol [2] describes an increasing “super-convergence” of technologies that is transforming the existing health care system into a digital health care system. The key characteristics of this new system are individualization, precision, and prevention. It is expected that this will result in data-based health care that will be characterized by a pronounced intensification of interdisciplinary cooperation and a stronger participatory role for patients. Every patient is increasingly becoming a “big data” challenge, with huge amounts of information about previous illnesses and conditions. At the same time, existing medical knowledge is growing exponentially. These two facts cumulate in increasingly complex decision-making processes in patient care. One recent digital transformative technology that can help bridge this complexity gap by preparing, analyzing, and organizing large amounts of data is artificial intelligence (AI). In health care, AI is becoming increasingly important for extracting and interpreting clinically useful information from large volumes of digital data and information sources and, in some cases, deriving recommendations for therapeutic action.

In the following section, the term “AI applications in medicine” refers to medical software, devices, and technologies such as apps whose analytical processes are AI-based and which are used in the health care sector by patients and/or practitioners.

### AI Applications in Medicine

Integrating AI applications into medical processes can automate repetitive tasks currently handled by humans. This hybrid working model improves human performance through technology. In 2012, the US Food and Drug Administration (FDA) certified a medical AI application for the first time [3]. Currently, the FDA database comprises 950 applications (as of the last FDA update on August 7, 2024), predominantly in radiology and the cardiovascular field [4]. Clinical AI systems have already demonstrated expert-level performance in radiology [5-7] and equaled the diagnostic performance of health care professionals in medical imaging [6]. Beyond radiology, there are numerous publications on the clinical application of AI [8-16] and large language models such as ChatGPT [17-19]. A scoping review by Han et al [20] generated an overview of all published randomized controlled trials on clinical AI as of November 2023 and found 84 studies. Their review underpins the growing evidence for the use of AI-supported tools in health care. However, from a populational and thus patient perspective, attitudes toward AI in health care are still fluid and demonstrate varying levels of knowledge, acceptance, and skepticism across different countries and demographic groups [21-24].

### The Need for Curricular Training About AI in Medicine

This development has far-reaching implications for doctors and requires a fundamental examination of AI systems [25,26]. At present, neither medical professionals already practicing nor the generation currently studying is adequately prepared for the integration of AI in medicine. At the same time, both groups will—or are already—encountering actionable AI in their day-to-day work that is or will be able to predict, diagnose and, if necessary, treat diseases [2]. At a clinical level, doctors require the competencies to critically assess AI applications to use only those tools that have an evidence-based effect on improving clinical workflows or patient outcomes. At the development level, it is also important to ensure that doctors are actively involved in the development and scientific testing of new AI applications. This raises the question of the extent to which these systems can be effectively integrated into the diagnosis and treatment process, as well as how limitations of the systems can be recognized by medical users and how a fallback level can be ensured. In rapidly changing health care systems, it is therefore essential to ensure that doctors have the knowledge, skills, and attitudes to both master current challenges and be prepared for future challenges [1].

The basic competencies required for this must be learned by medical students during studies and continuously developed throughout their careers [27]. There are already various international ideas for this qualification mandate. For example, the Standing Committee of European Physicians addresses this goal in its Policy on Digital Competencies for Doctors and defines digital core competencies [28]. The EU Health Policy Platform has formulated specific instructions for achieving these core competencies [29]. According to these policymakers, educators should consider including content about the following skill sets into their curricula: (1) general digital skills (data and software security, ethical and legal implications), (2) technical digital skills (telemedicine, AI, health apps, smart devices, robotics, virtual reality/augmented reality, data literacy), and (3) the patient-doctor relationship (digital communication and collaboration, digital health literacy). Seth et al [30] created a theoretical framework of topics related to AI that need to be taught to train medical students in this technology. Laupichler et al [31] emphasize the need to assess medical students’ AI literacy and attitudes in order to hone medical curricula to the AI educational needs of the next generation.

According to a recent review by Gordon et al [32], a growing number of medical schools are addressing AI throughout medical studies, but this is limited by the fact that only 2 of the 278 included studies focused on educational competencies in AI. For the German landscape, a study on national course programs found that the majority (72%) of surveyed medical schools stated that they offer AI-related learning opportunities [33]. In contrast to this, 70% of German medical students indicated in a survey conducted at the same time that they had never received any education in digital topics [34]. This surprising discrepancy can be explained by the fact that, although most German medical schools report offering such opportunities, they are mostly part of elective or extracurricular courses, with only 2 institutions including a separate subject specifically on AI in the core curriculum. As a result, existing AI curricula are currently only available to a very limited number of students, and large-scale AI education is still lacking. Recent studies consistently highlight knowledge gaps in AI education from the perspectives of both international medical students [31,35-37] and German medical students [31,34,38-41]. Students displayed low familiarity with AI and limited awareness of its potential applications in health care; they also reported limited or uncertain access to AI education in medical training. However, they believed AI training would be beneficial and showed great interest in working with it. Results differed regarding attitudes. Although Alkhaaldi et al [36], Moldt et al [42], and Laupichler et al [31] found students to be more optimistic and accepting about AI applications, in Boillat et al’s [37] survey there was more skepticism among students regarding the potential harm of AI for patients and job safety within the medical profession.

Despite the increasing number of medical schools that include AI-related teaching according to recent literature, current medical curricula struggle to meet the demands of students to equip them with a strong competency base to interact with, integrate, and critically evaluate AI tools in their clinical practice. Integrating education on core AI competencies into the general curriculum on a broader scale could significantly improve students’ experience levels with AI, enhance their attitudes toward the technology, and better prepare them to navigate medical AI effectively in clinical practice.

The objective of this paper is twofold. The first part introduces the concept of a novel, multisession elective course, “Medicine in the Digital Age,” which integrates AI teaching in the context of digital transformation into the medical curriculum at a German university. The second part presents findings from a qualitative interview evaluation of participants’ feedback on the AI module as well as their overall experience of the elective. Our aim was to conduct an explorative prospective study using a semistructured qualitative interview approach to generate a multidimensional insight into students’ knowledge, skills, and attitudes in dealing with AI in medical practice and their overall learning experience. For this, we asked students to comment on the following a priori deductive dimensions of interest: “Areas of AI Application,” “Future Work,” and “Critical Reflection.” The interview findings supported the iterative refinement of our teaching concept, as well as the ongoing educational reform processes.

## Methods

### Ethical Considerations

The local ethics committee was consulted during the development of the teaching evaluation for the curriculum presented. Following the consultation, the committee confirmed that the teaching evaluation constituted an additional quality assurance measure for teaching and curriculum development. This evaluation complements the existing concept for quality assurance at the Mainz University Medical Centre [43]. In accordance with the committee's recommendations, it was determined that an ethics vote was not considered necessary. However, participation in the evaluation required informed written consent from all students involved and was strictly voluntary. No compensation was provided to participants. To ensure confidentiality, all responses were pseudonymized prior to analysis.

### Structure and Rationale Behind the Module on AI

The teaching module “Artificial Intelligence” is part of the competency-based multisession elective course entitled “Medicine in the Digital Age.” The overall course aim is to equip students with digital skills that can be applied in a medically sound, technically feasible, legally compliant, data protection–compliant and ethically responsible manner and thus prepare them for the working environments of the future [2]. It was the first curriculum of its kind in Germany [1].

**Figure 1.**
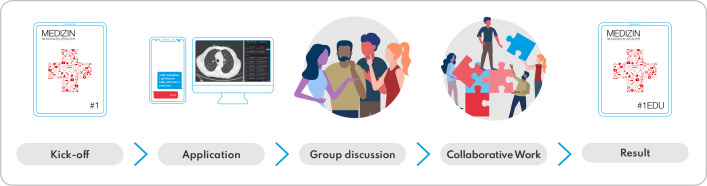
Didactic concept consisting of e-learning, workshops, discussion/reflection formats, and transfer. The results of the learning process are incorporated into the e-learning e-book.

The “Medicine in the Digital Age” course consists of 5 modules: AI; digital doctor-patient communication; digital health applications and smart devices; telemedicine; and virtual/augmented reality and robotics. The course is offered to medical students in their clinical years as a 5-day elective, with each module comprising an 8-hour face-to-face course day. The didactic concept follows a flipped classroom and blended learning format by combining e-learning (e-book), face-to-face teaching (hands-on workshops, practical exercises, discussion and reflection formats), coproduction, and transfer projects (Figure 1). The different formats alternate over the course and build on each other.

Using an interactive e-book, the participants deal with topics of digital transformation in the preliminary stages of the course. The e-book was created by our working group consisting of experts from medicine, medical education, ethics, media education, data science, and data protection, and also included patient perspectives. Its content mirrors the program of the elective course and contains a dedicated chapter for each module, combining theoretical background, reflective articles, and stakeholder or patient interviews. Students are expected to read the corresponding chapter in preparation for each course day to independently develop the basics of digital medicine. The entire e-book follows the collaborative concept of “Do it by the book, but be the author” [44]. By incorporating all student transfer projects into an iterative version of the e-book, students are encouraged to actively interact with the course and become the “authors” of their elective course’s e-book after completion of the elective.

The thematic breadth and interconnectedness of medical specialties necessitates an interdisciplinary team of lecturers. Therefore, onsite teaching is carried out by various medical disciplines (anesthetists, surgeons, medical informaticians, psychologists, pediatricians, psychosomatics, radiologists, orthopedic and trauma surgeons). In addition, computer scientists, representatives of federal state data protection and medical ethics, and patients complement the team of lecturers in the spirit of a transdisciplinary approach.

For the AI module, e-learning combined with face-to-face teaching and transfer tasks results in a total of 20 hours of teaching on AI. In accordance with the KSAVE model (Knowledge, Skills, Attitudes, Values, and Ethics) [45,46], the AI module aims at teaching the following overarching competences:

1. The student can describe various areas of application and programs that work with AI and is able to categorize clinical AI assistance systems in medical treatment in an evidence-based manner.
2. The student is able to explain examples of AI-assisted anamnesis, clinical examination, diagnosis, and therapy.
3. The student is able to name limitations of AI applications in current clinical practice and to evaluate the use and benefits of AI within the complex interplay of technical, legal, and ethical principles as well as under sociopolitical framework conditions and to place them in a medical context.
4. The student is able to reflect on how roles in the medical profession will change or evolve in the light of integrated AI assistance systems.

To give an example: a student demonstrates competence mastery by reflecting on the integration of AI-based systems into clinical workflows in oncological imaging in radiology, emphasizing their role in improving early identification of curative versus palliative needs and complementing clinical decision-making. They critically evaluate practical applications across anamnesis, diagnosis, and therapy, addressing limitations such as data quality, clinician acceptance, and ethical concerns. Through this analysis, the student links theoretical knowledge to real-world challenges, demonstrating readiness to apply AI to improve patient care and health care processes.

**Figure 2.**
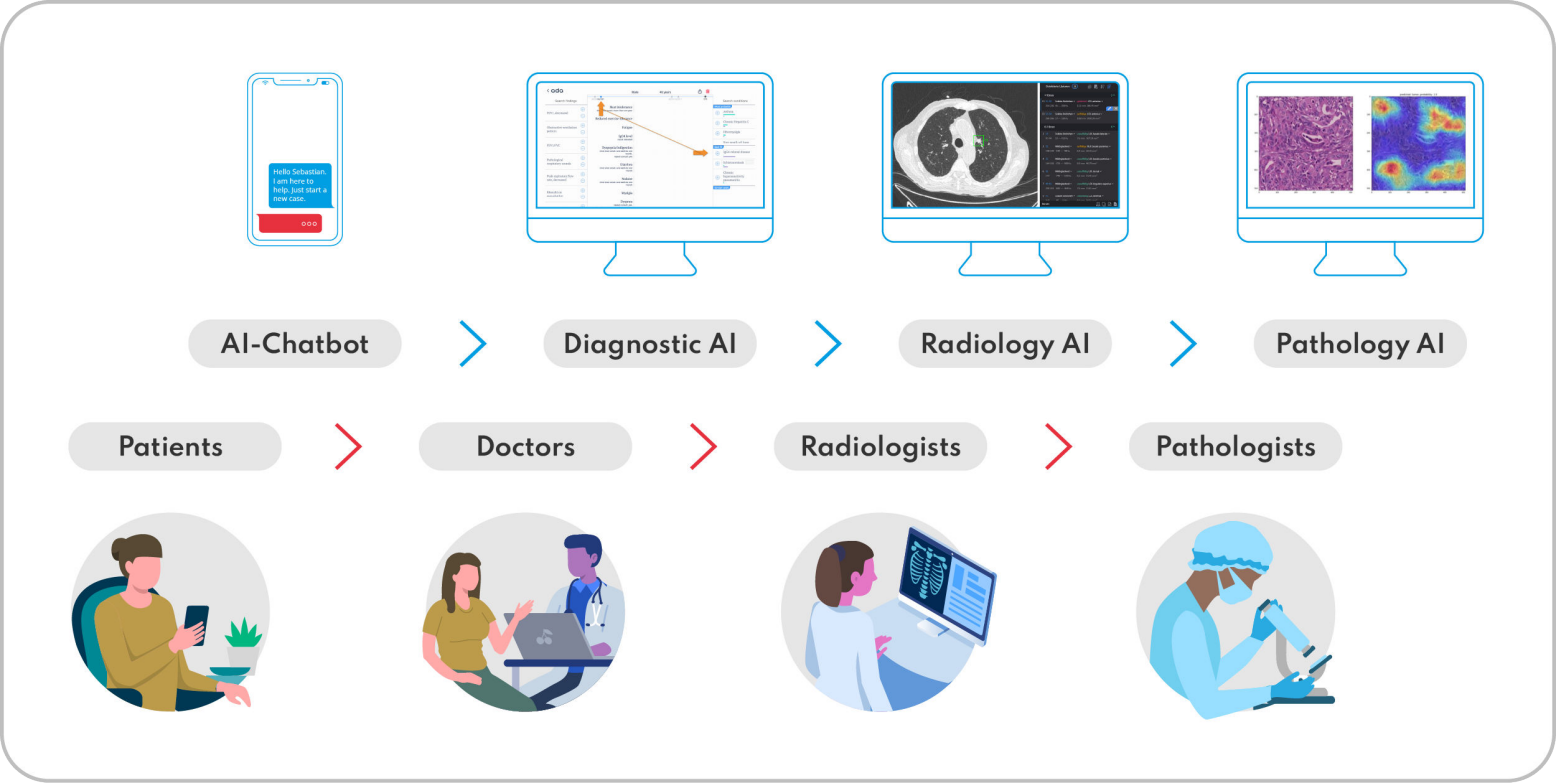
Workshops within the AI curriculum demonstrate hybrid workflows between humans and AI. The human workflow (lower section) is enhanced by the integration of AI-based narrow intelligence (upper section) along the patient care continuum by various medical specialists. AI: artificial intelligence.

In the onsite teaching of the AI module, the focus is on practical workshops (Figure 2). These are designed to illustrate the integration of AI into medical treatment processes, followed by discussion and reflection sessions to promote the transfer to the students’ own actions.

### Workshop on Medical History/AI Chatbot

To address the relevant technologies (ie, natural language processing, large language models, and chatbots), students are introduced to an AI-based smartphone app (Ada Health), which acts as a chatbot to take a symptom-based clinical history and make a suspected diagnosis [47-49]. Students then take a clinical history in groups of two from one of the lecturers, who takes on the role of a patient based on a predefined case vignette. First, one of the students takes a classic medical history and formulates a suspected diagnosis. Thereafter, the second student obtains a medical history of the same patient case by reading out the chatbot’s questions. Subsequently, the independently formulated suspected diagnoses are compared with the suspected diagnoses of the chatbot in the entire group. The students discuss which anamnesis questions and diagnoses they did not consider, and which questions and diagnoses the chatbot did not list. The usefulness of the different suspected diagnoses is then discussed and differences in the clinical histories are explored to determine the advantages and disadvantages of each method.

### Workshop on Radiology/AI-Supported Radiology

Together with a radiologist, students learn how digitalization is changing the way radiologists work (eg, Picture Archiving and Communication System, radiology information system, speech recognition). Radiological AI applications are demonstrated as examples. Specifically, an AI for the automatic detection of tumor-specific lung foci is demonstrated. The software (InferVision, InferRead CT Lung) provides the user with the size and localization of the lesion as well as an estimate of the malignancy as a percentage. Additionally, an AI application for the automatic diagnosis of conventional X-ray examinations of the thorax is presented (Oxipit, ChestEye). The students learn that a number of published papers have already shown that various AI applications are equivalent to radiologists in individual subtasks [6].

### Workshop on Pathology/AI-Supported Pathology

Students are introduced to the influence of digitalization on the field of pathology. For this purpose, a pathologist demonstrates the use of AI as a supporting tool in the diagnosis and detection of malignant changes in histopathological tissue sections [16]. Both the technical and informative background, as well as the process of developing and scientifically evaluating an AI application (AI development life cycle) and its practical application (integration into patient care), are illustrated. Students learn more about the future potential of AI in pathology and can ask questions and contribute their own thoughts.

### Discussion and Reflection Formats

For reflection, students and lecturers discuss the following questions together in fishbowl discussions:

- What are the opportunities and risks of using AI in the context of patient treatment?
- How do we deal with probabilities calculated by an AI?
- What will your day-to-day work look like in 2025?
- What new skills will you need in the future?

### Transfer Projects

Throughout the course, students work in self-selected small groups (groups of 4) on the overarching task of researching a useful medical AI application and presenting it in plenary on the last day of the course. The group presentations are followed by 15-minute discussion rounds with the plenum. The transfer projects students chose reflect the wide range of AI in terms of technology (language, imaging, data procession), medical use cases (conservative medicine, surgical medicine), and different age groups (from AI solutions for pediatrics to palliative care). In addition to their research, part of students’ transfer performances also lies in critically analyzing and presenting their various solutions. Among others, students addressed topics such as an AI-supported ultrasound image navigation for regional anesthesia [50], an AI algorithm supporting clinicians in sepsis management [13], or an AI algorithm predicting the end of life developed by Stanford University [51].

### Evaluation

**Figure 3.**
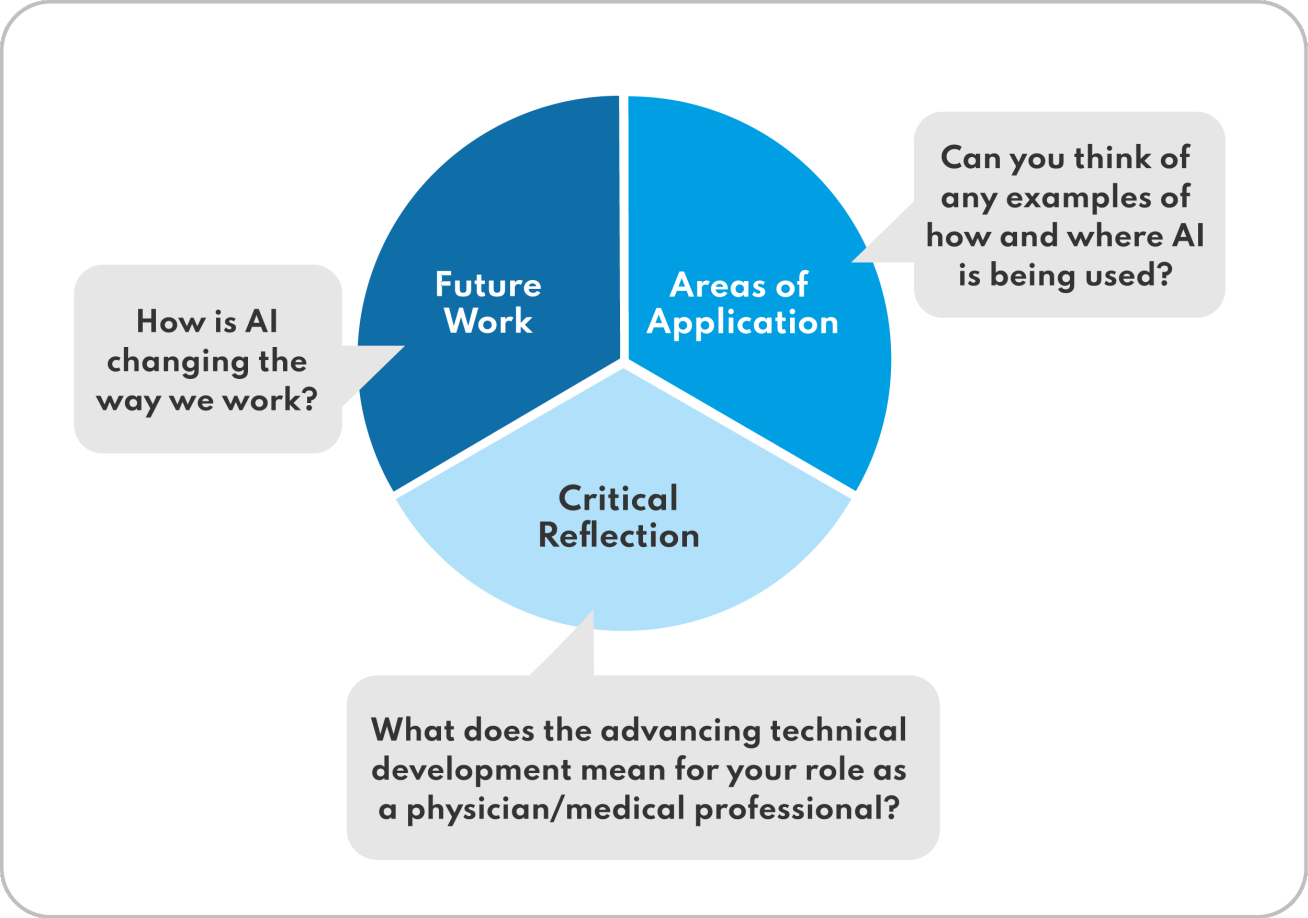
Outline of the main points of the interview guide questions on AI (circle) including guide questions (boxes outside). AI: artificial intelligence.

The elective course on Medicine in the Digital Age including the presented AI module was introduced to the medical curriculum at the University Medical Centre of the Johannes Gutenberg-University Mainz. The evaluation of the 3 groups consisting of medical students in their second and third clinical year (ie, years 4 and 5 of the 6-year medical program) was carried out using semistructured, focused, guided group interviews consisting of open and targeted questions based on Merton, Fiske, and Kendall [52,53]. The interview questions were formulated based on the KSAVE model. The interview guide aimed to ascertain the participants’ existing competencies in the areas of knowledge, skills, and attitudes in dealing with AI in physicians’ practice. This theoretical background resulted in 3 main interview topics: “Areas of Application,” “Future Work,” and “Critical Reflection” (Figure 3). Additionally, the last section of the interview addressed students’ overall assessment of the course. The interview guide was used to evaluate the entire course and is provided in the appendix (Multimedia Appendix 1).

**Figure 4.**
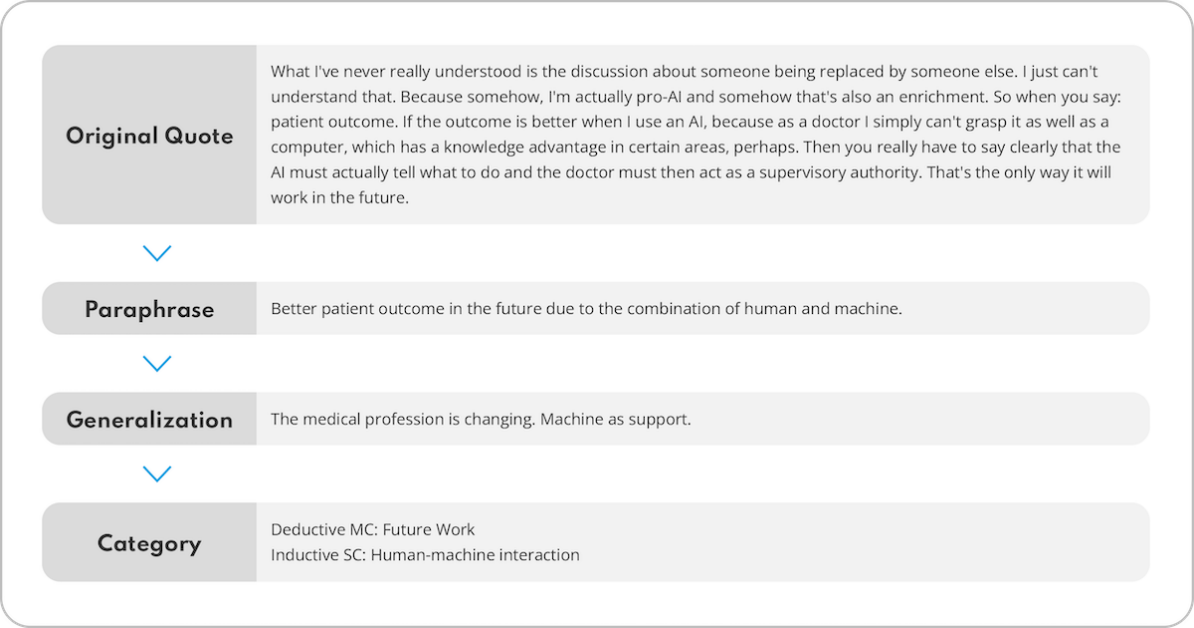
Process of category development through the continuous comparison of the content of the deductive categories with the compiled material. Through the steps of paraphrasing, generalization, and reduction of the content-structuring content analysis, additional inductive categories can be developed, and the statements can then be assigned to an explicit category. MC: main category; SC: subcategory.

The focus group interviews took place within 2 weeks of the comprehensive course. Participants were informed about recording, transcription, data usage, storage, and privacy, and consent was obtained beforehand. The interviews were conducted and recorded by a researcher with expertise in qualitative research. The audio files were then transcribed for further analysis by 3 student research assistants (KD, LU, and EK). The interview transcripts were subsequently analyzed using content-structuring qualitative content analysis according to Mayring [54]. This is a text analysis method that follows a logical, systematic pattern and aims at transferring raw text data into structured categories (Figure 4). Categories can be formed deductively based on previous knowledge or hypotheses, or inductively based on new, text-immanent findings. For a structured evaluation of the results, the categories formed in the category system were organized hierarchically into main categories (MCs) and subcategories (SCs). The MCs were deductively derived from the research questions prior to the survey phase. During the analysis process, additional inductive SCs were formed from the interview statements. Inductive coding was used and codings were discussed and agreed upon by the coding research assistants. Saturation was achieved by interviewing all participants and subsequently coding all interview material.

The Results section presents the parts of the overall evaluation results that explicitly relate to the AI module and the overall course assessment.

## Results

### Evaluation Outcomes

From 3 group cycles, 18 semistructured, focused, guided interviews were conducted with all 35 participants (female=11, male=24) from the 3 consecutive courses, which formed the basis for the qualitative evaluation of the course concept. The interviews lasted 24:36 minutes on average. In all interviews, a total of 214 statements were made that could be assigned to the area of “Artificial Intelligence” and 610 statements related to “Overall Course Assessment.”

**Figure 5.**
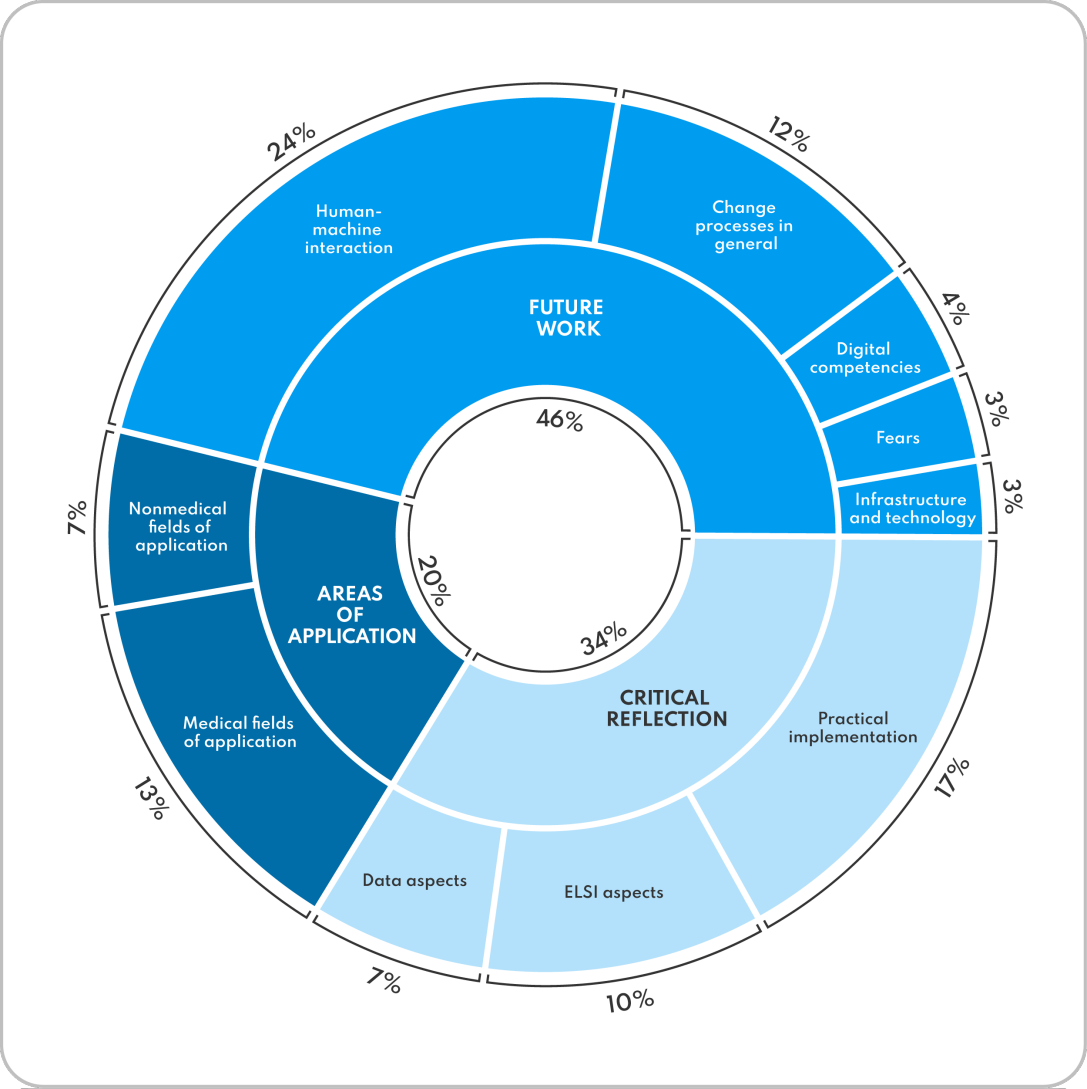
Graphical depiction of the qualitative research results on the AI teaching module broken down into main and subcategories, based on 214 coding units (student statements). Percentages are relative to the overall sample of 214 statements on AI. All percentages are rounded. AI: artificial intelligence; ELSI: Ethical, Legal, and Social Implications.

Following the analysis steps outlined above, the 3 deductive MCs related to AI—“Areas of Application,” “Future Work,” and “Critical Reflection”—were assigned further concrete inductive SCs derived from the content of the text during the evaluation process (Figure 5). Statements in the MC “Overall Course Assessment” were divided into the 6 SCs “Learning Experience,” “Learning Success,” “Structure,” “Content,” “Methods,” and “Conclusion.” For quality assurance purposes, the results report was written in accordance with the Consolidated Criteria for Reporting Qualitative Research (COREQ) checklist [55]. The following section details the qualitative results for each main category. Anchor quotes support the result report for each category. For this, quotes were translated into English by the authors and minor changes were made to improve readability. An extensive overview of anchor quotes for all SCs is provided in the appendix (Multimedia Appendix 2). Identification codes in parentheses accompany each quote to allow allocation to individual participants (the ID code reads as follows: course run:interview number:speaker ID).

### Main Category “Areas of Application”

This category investigated students’ knowledge about the existence of different AI applications in health care and beyond and their attitudes toward them after completing the elective course. A total of 20% (43/214) of all statements on AI fall into the category “Areas of Application.” Of these, 29 fall into the SC “Medical fields of application” and 14 into the SC “Nonmedical fields of application.”

For the SC “Medical fields of application,” students listed a variety of medical use cases. The topics that were covered in the course dominated, namely diagnosis in general as well as image diagnosis in radiology, pathology, and dermatology.

During the week, for example, I learnt that the radiologists can have the lung round foci assessed by AI during the CT scan. With different probabilities. […] Then we learned in the pathology department that in the future AI will also calculate […] how high the probability is whether it is a tumor or not. Or […] this dermatology AI, which can show whether it’s a melanoma or a benign mole. […] So I’ve definitely learnt a lot, I could go on listing all the examples.

[3:6:F]

In terms of diagnosis, participants rated the use of AI in triage and preliminary anamnesis in the outpatient sector as useful. This could reduce waiting times and counteract missing information. Regarding image diagnosis, the students refer to AI as a “safety net” backing up their own findings with a digital second opinion.

Interviewees expressed disbelief about the status quo on several levels. They discussed the limited usage of AI applications in everyday clinical practice and their own lack of knowledge before attending the course. They identified a general absence of curriculum focused on AI in medicine within their own course of study. Overall, students were surprised by the variety of possible applications, as well as by the rapidly increasing number of market-ready AI medical products. They expressed great interest in the use of AI in their medical practice and see the meaningful and context-specific application of AI as an urgent task of the present.

For the SC “Nonmedical fields of application,” at the nonmedical level, students mentioned various possible or already existing application scenarios for AI, some of which have been adopted into everyday life without reflection (eg, voice assistants, navigation, purchase recommendations). The most attention was paid to autonomous driving and transportation.

### Main Category “Future Work”

A total of 46% (99/214) of all statements on AI fall into the category “Future Work.” Of these, 51 fall into the SC “Human-machine interaction,” 26 into the SC “Change process in general,” 9 into the SC “Digital competencies,” 7 into the SC “Fears” and 6 into the SC “Infrastructure and technology.”

For the SC “Human-machine interaction,” students focus on the question of how AI can contribute to becoming a better doctor. The students state that they thought intensively about the combination of humans and machines during the course week and consider this combination to be the optimum for the future practice of medicine.

Just like the chess player and the computer together (Centaur Chess computer), they are unbeatable. […] and hopefully it will be the same with the doctor [and AI].

[2:1:3]

“Shared decision making” and digital second opinions can counteract incorrect treatment or improve treatment outcomes in the sense of assistive systems and thus increase patient safety. The students describe their experiences with the anamnesis chatbot and characterize the comparison of the two forms of anamnesis as very informative. The differences between the chatbot anamnesis and their own anamnesis practice were thus revealed. Emphasis was placed on the aspect of social anamnesis, which the students carried out more intensively than the chatbot. However, the students rated the chatbot’s anamnesis procedure as more structured and systematic than their own. Students predominantly regard AI as an opportunity.

For the SC “Change processes in general,” students did not consider the medical profession to be threatened by the use of AI, but the practice of the profession and the subdivision of specialties may change. Students stated that the rapid increase in knowledge means that doctors are even more obliged than before to undergo continuous further training. The preinformed patient will become more of a discussion partner at eye level. The interviewees see this as a great opportunity to improve the doctor-patient relationship, as the inclusion of AI in routine medical procedures could lead to an increase in personnel and time resources, allowing doctors to focus on the traditional core medical activities of consultation, treatment, and care.

For the SC “Digital competencies,” the competency profile and requirements for the medical profession are also changing as a result of the transformation. Here, the interviewees speak of a lack of or inadequately trained digital skills, which are also not considered in the standard curriculum. Students would like a safe framework for trying out new technology. Furthermore, the use of AI in everyday medical work requires clear quality criteria that are similar to a review process for evaluating technologies. The ability to correctly classify AI-generated information and to critically question the statements made by the AI is regarded as particularly relevant. According to the students, it is and remains the task of the doctor to assess which application is appropriate for the individual patient and when treating physicians need to actively decide against its use.

Where does the AI get the data from? How is it analyzed? And above all, how is it checked to make sure it really is a sensible AI? You need to know that. In a way just like we learn how to read scientific publications. And decide whether they are good or bad.

[2:1:3]

For the SC “Fears,” the biggest problem addressed is general ignorance and the resulting fear of, for example, the threat of job losses. Students suspected that this fear is the reason why AI applications are often not developed by health care experts, but by fast-moving commercial companies. Students also considered the combination of human and machine to be problematic if it is not possible for the doctor to understand how the AI operates and reaches decisions.

For the SC “Infrastructure and technology,” the students note that the technical change in everyday working life is particularly noticeable through deficits in (technical) equipment.

### Main Category “Critical Reflection”

A total of 34% (72/214) of all statements on AI fall into the category “Critical Reflection.” Of those, 36 fall into the SC “Practical implementation,” 22 into the SC “ELSI aspects” (Ethical, Legal, Social Implications), and 14 into the SC “Data aspects.”

For the SC “Practical implementation,” students see opportunities for larger-scale, international cooperation. This requires openness and investment in progress and research. They take their practical experience from the “clinical anamnesis” workshop as an example of low-threshold contact, which they want to take out of the course to raise awareness. The limitations of the chatbot, for example, making a misdiagnosis, are critically questioned. In such cases, the intended time saving backfires and becomes extra work.

That would unsettle me […] if something completely different comes out as a treatment suggestion or what I see in an MRI image or something like that, then it would make me very insecure and then I would want to make sure. Be it through the senior physician or that I can just have a look: How does this AI come up with this? And if that doesn’t work, then it’s unfavorable for the procedure. Then you have to rely on the senior consultant again and maybe the head physician […] and then I’m back where I was before without AI.

[2:1:3]

One problem discussed is that current AIs can only act as “narrow intelligence” in very specific settings, meaning that anomalies that do not correspond to the AI’s specific field of action remain undetected. This results in the risk of unconsidered use. In general, all interviewees share the opinion that not everything that is technically possible should also be used in practice and that each use case must be considered individually.

For the SC “ELSI aspects,” the question of whether increasing digitalization will reduce or intensify doctor-patient contact is viewed critically. On the one hand, digitalization represents an opportunity to relieve doctors and invest the freed-up capacities in the doctor-patient relationship. On the other hand, there is a risk that AI will impact the interpersonal interaction and thus the patient’s individuality.

The possibility of consciously influencing AI is the subject of intense ethical debate. Specifically, it is questioned at what point it is unethical not to use the advancing technology, as this would deliberately deny the patient the best possible treatment.

At some point it becomes unethical not to use such things. […] That’s actually the point. Why are we always so afraid that we’re not important enough? At some point, the doctor is no longer the all-knowing person.

[2:3:1]

The interviewees see a further ethical dilemma in the case of a discrepancy between the diagnosis provided by the doctor and the AI. The right not to know and the handling of probabilities play a decisive role in sensitive areas, such as prenatal or genetic diagnostics and palliative medicine. Students also discussed the unclear legal situation regarding liability issues as a possible cause of rejection of AI applications.

For the SC “Data aspects,” regarding data protection, too little regulation violates personal rights. Too much regulation makes it difficult or, in the worst case, prevents access to data for clinical research. In general, students also question the lack of traceability of AI results. They critically note that convenience or lack of time can lead to the results not being checked over time.

### Main Category “Overall Course Assessment”

Of all 610 “Overall Course Assessment” statements, 134 fell into the SC “Learning Experience” and 108 into the SC “Learning Success.” The remaining statements were categorized into the SCs “Structure” (n=61), “Content” (n=126), “Methods” (n=142), and “Conclusion” (n=39).

For the SCs “Learning Experience” and “Learning Success,” students highlighted engaging with AI and digitalization as a significant learning success, given the absence of such topics in the standard curriculum.

I'm just glad that I had this week, because it really showed me what we don’t learn at university. And how big the topic actually is for us.

[2:2:2]

They described the hands-on interaction with various technologies as “eye-opening*”* (2:2:4) and the group work on human-AI comparisons as “impressive” (2:1:4). Many students, initially skeptical or ambivalent about AI, reported increased knowledge and awareness of AI technologies and a deeper understanding of their impact as a result of the elective. They felt better prepared for their future careers regarding questioning and categorizing digital tools such as apps or AI, and underlined the gain in competences:

I think everyone left with a gain in expertise. Be it in the form of medical expertise, technical expertise, or simply that you’ve thought about things like data protection and apps and so you’ve also gained absolute everyday expertise.

[1:3:B1]

For the SCs “Structure,” “Content,” and “Methods,” students appreciated the involvement of diverse experts, valuing the variety of perspectives on the technology.

What was outstanding […] was that the input came from the legal side, from the ethical side, from the technical side somehow every time.

[3:8:A]

They praised the active and innovative learning format of the elective, noting that it encouraged reflection and engagement rather than the rote learning typical of other subjects.

It’s often the case that you’re told things and then you have to memorize them. And here it was more the case that you were given information but then had to think about it yourself, for example to discuss it or draw a picture or whatever. And that’s a completely different kind of learning, which unfortunately we don't usually do that much of in our degree programs. So I thought it was really good. Because these are actually skills that you should have and not that you can somehow memorize a book.

[2:1:3]

The discussion formats were highlighted as a distinctive feature in comparison with previous teaching experiences, with critical reflection helping students develop a more nuanced understanding of the topic.

I think you learnt an incredible amount, especially in the discussions, and you were actually forced to really think about certain theses. I also found this kind of discussion extremely productive.

[2:2:4]

For the SC “Conclusion,” students almost unanimously agreed that the elective had broadened their horizons and appreciated the opportunity to participate. They wished that the course would be expanded so that more students could participate. Some expressed a wish for more breaks or even longer discussion sessions. Although students felt that the scope and time commitment of the elective was appropriate, many would have liked it to last longer:

“I think the biggest minus is actually the time. It’s rare that you leave a course saying: ‘Hey, I wish I’d stayed longer.’ But […] Tuesday and Wednesday were actually days when I thought: ‘Okay. I could have stayed two hours longer’.

[2:2:5]

In summary, the qualitative evaluation showed a high level of acceptance of the course concept and differentiated attitude toward AI among students. The course participants emphasized the increase in their knowledge and competences about the technology as well as the appreciation they felt as a result of the intensive and varied collaboration with each other and with the lecturers. The opportunity for critical discussion, practical interaction, and application was rated particularly positively. All 35 students stated that they would recommend the elective course to peers.

## Discussion

### Principal Findings

The digitalization of medicine and the use of AI applications is a fundamental process of change that will have a major impact on the future job profile of doctors to an extent that cannot yet be foreseen. What is certain, however, is that we are transitioning from the “information age” to the “age of artificial intelligence” [26] and that the integration of AI into medical treatment processes will redefine human-machine interaction. It is therefore essential to prepare future doctors to use AI in daily practice [27]. At present, although curricula are beginning to change, structured teaching concepts are lacking in terms of curricular mapping, although educators and practitioners emphasize the need to impart such competencies both nationally and internationally. Most students also advocate for AI education in their studies and report limited or no exposure to AI technologies and learning resources [35-37,41]. This does not mean that students must be able to program themselves but they must learn the practical application of AI in line with ELSI principles, data science, biostatistics, and evidence-based medicine during their studies [30,56].

The qualitative results of the AI module show that the embedding of curricular teaching about AI is generally feasible and sensible, that the added value of such a teaching module is recognized by students and acknowledged with great interest and acceptance, and that it leads to an increase in competence among students and promotes a critical and reflective attitude toward new technologies. Regarding the core aspects reflected in the main categories of the analysis (Areas of Application, Future Work, Critical Reflection), several key points can be learned, as detailed in the following sections.

### Areas of Application

At present, it is not sufficiently clear how and when AI should be used in clinical diagnostics and therapy. Regarding the “how,” students demonstrate a forward-thinking and nuanced examination to potential AI applications in clinical settings.

With regard to the “when,” clarification is needed on the specific areas and questions where AI can assist in the clinical workflow [30,57]. Here, students express ambivalence about its integration, acknowledging both benefits and risks.

### Future Work

Regarding patient care, students highlighted AI’s potential to enhance care through personalized application and resource optimization, aligning with its reported ability to save time and personnel resources amid health care resource scarcity [58]. Students expressed some apprehension about the future impact of AI on the medical profession, though concerns about career choices were less prominent. This aligns with a survey in which 83% of medical students disagreed that AI would render radiologists obsolete [41]. Nonetheless, a minority of participants expressed fears about career prospects. Although AI’s full impact remains unpredictable, it is undeniable that medical professions will change. Wartmann and Combs [26] speak of a “reboot” of the health care system and postulate the need to skillfully manage the interface between medicine and machines, as AI will surpass human capabilities in certain tasks [26]. Reflecting this, students stressed the importance of human-machine interaction and corresponding digital skills.

### Critical Reflection

Most students underlined the potential of AI for their future career while maintaining a critical perspective, avoiding blind enthusiasm. They emphasized the risk of AI manipulation and its consequences for patient care, underscoring the need for doctors to retain ultimate decision-making authority over AI recommendations. The evaluation presented here thus indicates students’ development of a critical attitude due to the module. These findings underscore the importance of future medical curricula teaching students to integrate AI assistance into their decision-making processes [2].

At the industry and developer level, students also acknowledged the need to design AI applications with ELSI aspects in mind. Such recommendations already exist. For example, the multisociety statement on the ethics of AI in radiology [59] and a white paper from the European Society of Radiology outline key ethical and practical considerations for the responsible use of AI in clinical practice [60].

In summary, students acknowledged the evolving nature of AI in health care as well as the necessity for skillful management of the interface between medicine and AI. They emphasized the importance of human-machine interaction as well as the need to develop digital skills while maintaining a reflective mindset toward technology.

### Implications

Current literature on medical students’ evaluation of their AI competencies demonstrates a relevant knowledge gap and the need for rapid-employment curricula solutions to change this. Overall, both the Medicine in the Digital Age elective as well as its AI teaching module were demonstrated to be feasible and reasonable teaching concepts, which supported maintaining the blended learning approach and the basic content of the modules. Nevertheless, valuable insights for iteration were drawn from the evaluation. First, the course has been updated to reflect technological and regulatory developments, such as the AI Act. Second, insights from students’ transfer projects and reflective discussions informed an “agility by design” [61] approach, incorporating noteworthy projects or themes identified through students’ input into the subsequent course iterations.

With the didactic framework, course design, and content outlined, this teaching concept can serve as a transferable model for implementation and adaptation in other universities or training settings. Adjustments may be required to address specific target groups or local circumstances.

Evaluating the AI module’s learning objectives—knowledge, skills, and reflection—is critical in both simulated (eg, Observed Structured Clinical Examination exams) and real-world settings. Complex educative interventions like this require robust assessment of efficacy and sustainability. Future research should explore whether a single elective suffices, if refresher courses are needed, or if phased AI education is beneficial. To validate this teaching course, prospective longitudinal trials comparing students who attended the AI module and untrained students are essential.

### Methodological Strengths

The “Medicine in the Digital Age” curriculum described here addresses the digital transformation of medicine in an interdisciplinary and interactive way for medical students. AI is one of the 5 teaching modules and the rapid development and adoption of AI technologies in health care requires students and professionals to familiarize themselves with it and develop an attitude toward it. Standard quantitative methods can only inadequately depict the development of a professional attitude. The potential of the qualitative methodology used in teaching research should therefore be emphasized. Qualitative approaches provide insights into the learners’ assessment of individual learning success, including gains in the areas of knowledge, skills, and attitude, as well as the content design and methodological structure. They are therefore ideal for the iterative further development of teaching concepts and the evaluation of attitude-oriented teaching content. The application of qualitative methodology represents a distinctive strength and unique contribution of this study. Although most research on medical students’ perceptions relies on quantitative questionnaire surveys, this study uses qualitative survey instruments. The 2 existing qualitative studies in Germany are limited by their focus on analyzing free-text survey responses [34] and by their narrow scope, specifically examining students’ attitudes toward mental health chatbots [42]. To the best of our knowledge, this study is the first to offer comprehensive, in-depth qualitative insights into German medical students’ perceptions and attitudes toward AI.

### Limitations

A common limiting factor in qualitative research is the small sample size. Helfferich [62] cites a sample size of between 6 and 120 respondents as appropriate. This means that our sample of 35 students can be assumed to have sufficient result validity. A second limitation could be that the results present a retrospective evaluation. Incorporating a qualitative pre-post analysis might have drawn a more concise picture of students’ changes in knowledge, attitudes, and reflection on AI as a result of the course. Third, the findings might not be generalizable to other medical training programs, student attitudes, countries, or demographics. Lastly, group dynamics in the focus groups might have influenced the outcome by introducing social desirability bias.

### Conclusions

Digitalization will continue to fundamentally change medicine. Therefore, in line with international appeals, today’s education and training curricula must teach students and practicing physicians the basic competencies for using digital tools such as AI applications. It is not enough to simply integrate context-specific AI solutions as teaching examples into existing curricula. The aim of future curricula must be to equip students with the key competencies for their future day-to-day work in the age of AI and enable them to internalize knowledge, skills, and attitudes toward these tools from the beginning of their training. As an outlook for the AI curriculum presented here, it can be said that it addresses this need in a unique way. The qualitative teaching evaluation showed that students were able to deal with the topic in a very differentiated way after the AI teaching unit. The transferability of the curriculum to other university locations can be assumed in principle. The curriculum could therefore serve as an exemplary teaching concept for other universities and contribute to training medical students in two future-oriented skills: AI literacy and its transfer to medical human-machine interaction.

## Supplementary material

10.2196/65220 Multimedia Appendix 1 Interview guide for artificial intelligence in medicine.

10.2196/65220 Multimedia Appendix 2 Qualitative results. Anchor quotes for all categories.

## Abbreviations

AI: artificial intelligence
COREQ: Consolidated Criteria for Reporting Qualitative Research
ELSI: Ethical, Legal, and Social Implications
FDA: US Food and Drug Administration
KSAVE: Knowledge, Skills, Attitudes, Values and Ethics
MC: main category
SC: subcategory
