# Supplementary material for: Novel Blended Learning on Artificial Intelligence for Medical Students: Qualitative Interview Study
Source: JMIR Med Educ. 2025 May 26;11:e65220. doi: 10.2196/65220 (PMC12149464; doi:10.2196/65220)
Supplement: Multimedia Appendix 1 [file mededu-v11-e65220-s001.doc]

**Multimedia Appendix 1.** Interview Guide AI in Medicine

**Interview Questions: Micro level**

What were your experiences with ADA?

What is your position on artificial intelligence?

What does the advancing technical development mean for your role as a doctor?

What does the advancing technical development mean for the patient?

**Interview Questions: Macro level**

Where do you see challenges and opportunities in this development?

Can you think of any other examples of how and where AI is being used?

What do you understand by big data?

How is big data changing the world of work?

Where do you have ethical/moral concerns?

**Interview Questions: Closing**

What is your overall assessment of the course?

What did you miss in the teaching concept?

Has your interest in the digitalization of medicine increased as a result of your participation?

Is there anything else you would like to say?
