# Supplementary material for: Novel Blended Learning on Artificial Intelligence for Medical Students: Qualitative Interview Study
Source: JMIR Med Educ. 2025 May 26;11:e65220. doi: 10.2196/65220 (PMC12149464; doi:10.2196/65220)
Supplement: Multimedia Appendix 2 [file mededu-v11-e65220-s002.doc]

**Multimedia Appendix 2.** Qualitative Results – Anchor Quotes for all categories.

| **Main Category** | **Subcategories** | **Anchor Quotes (ID)** |
| --- | --- | --- |
| Areas of Application | Medical fields of application | During the week, for example, I learnt that the radiologists can have the lung round foci assessed again by the AI during the CT scan. With different probabilities. [...] Then we learned in the pathology department that in future the AI will also calculate [...] how high the probability is whether it is a tumor or not. Or [...] this dermatology AI, which can show whether it's a melanoma or a benign mole. [...] So I've definitely learnt a lot, I could go on listing all the examples. (3:6:F)  What impressed me during the week [...] is [...] how many things there are that are already in use. (2:1:3)  I found it [...] frightening how little you know about things that are basically ready for the market [...] as medical students – If anyone needs to know, it's the people at university and those who are about to graduate, but I was completely unaware of that. [...] things like the fact that an AI can diagnose X-ray images just as well as a radiologist with 30 years of professional experience. (1:3:B1) |
|  | Non-medical fields of application | Autonomous driving. So, keyword Tesla, Google and Apple, who also want to develop cars. Which works amazingly well in some cases but is also a bit scary as long as it hasn't been introduced across the board and accidents are still happening. (1:1:B)  I can well imagine that it could also be used in the military sector. (1:1:A)  Everything that revolves around advertising on the Internet has also been networked with AI. (1:2:B1) |

| **Main Category** | **Subcategories** | **Anchor Quotes (ID)** |
| --- | --- | --- |
| Future Work | Human-Machine Interaction | Just like the chess player and the computer together (Centaur Chess computer), they are unbeatable. [...] and hopefully it will be the same with the doctor. (2:1:3)  Competitive thinking between doctors and artificial intelligence (2:1:1)  So if you think about it, [...] there is no human being who knows everything that is written in these (medical) books for every subject, [...] and if there is simply a machine that knows everything [...] that you have forgotten or don't know or nobody on the ward knows, then the PC knows it. (1:2:B4)  If a doctor somehow had the opportunity to use artificial intelligence [...] where it has been proven to be beneficial for the patient, and the doctor does not use it, it is actually negligent on his part. And I would expect that from my doctor, and I think it's also okay for patients to expect that. (2:1:3)  Before I did the course, I was really critical of it, just because of the lack of knowledge, you are initially hostile towards everything new […], and then you think it will replace you. But the fact that it can be so valuable and support so many processes was something I hadn't realized without this [elective course]. (1:2:E)  I still see the danger that – I mean, the everyday life of a junior doctor is simply stressful – that in order to reduce your own workload, you then give the AI more competences – so that this symbiosis tends to fall away and as a doctor you think: Ah, I don't have time for this right now, I'll just let the computer do it and it will be right [...]. And then at some point it might not be right and then something goes wrong. (2:1:2) |
|  | Change processes in general | I'm afraid that it will be a big challenge for doctors if a patient comes in with a diagnosis that an app [...] has suggested and now wants to be tested for it. Then, of course, the doctor first has the problem that if I test the patient for it, I give him the feeling that it might be possible, even if it is very unrealistic from my point of view. So, if the patient […] says "Well, you've convinced me, I don't have this after all." But in the end it turns out that it was the way this app found out, then I sue my doctor. So it's a legal challenge, how do I protect myself in such a case? [...] I think that will be very challenging. (1:1:B)  I don't think the medical profession will die out. But I can well imagine that at least some specialties will change a lot. (1:3:B2)  I think the word transformation is actually quite appropriate. So I really believe that this is an upheaval on a very large scale that will affect all, all areas. (3:8:H) |
| **Main Category** | **Subcategories** | **Anchor Quotes (ID)** |
| Future Work  (Table continued) | Digital competencies | We young people are actually caught in the middle of this conflict, [we] are already excellent at dealing with it and really only need to be properly trained and can actually practice Medicine 4.0. (3:7:G)  I don't think we need to know the details of the AI's decision-making process, but I think we should be given some basic knowledge if we are to use it. (2:1:2)  Where does the AI get the data from? How is it analyzed? And above all, how is it checked to make sure it really is a sensible AI? You need to know that. A bit like we learn how to read scientific publications. And see whether they are good or bad. (2:1:3) |
|  | Fears | There is always a certain degree of skepticism because even great thinkers who are a lot smarter than me [...] are already afraid of artificial intelligence. Of course, it has its place somewhere, because it [...] has a benefit for many people. [...] But you always have to weigh up the costs and benefits. And the moment this AI is already doing things that the programmer doesn't understand, it has to be viewed with skepticism. So, I really don't know where to position myself, how to position myself. I have to say, the event made me feel more positive. But I'm still skeptical. (1:3:B3)  Personally, I could imagine that I would find it difficult to use an AI if I didn't know exactly how it works. (2:1:3)  What is actually still a bit scary is that you no longer know how things work at all. (1:2:X) |
|  | Infrastructure and technology | Simply for yourself, so that if you have a practice at some point, for example, you don't start with the system of having one patient letter here, one report there. Instead, you need to find something that contains everything at once. That you make it as easy as possible for yourself, even with the technology you have. (3:1:2)  Actually everything that could be fed with images could be revolutionized in many places. (3:3:A)  It would also be practical if the documentation were to be displayed directly on the PC at all stations so that everyone could read it. You wouldn't have any writing problems. (3:1:3) |

| **Main Category** | **Subcategories** | **Anchor Quotes (ID)** |
| --- | --- | --- |
| Critical Reflection | Practical implications | Well, I see it more as-, not as saving time or anything like that, but rather as increased safety. (3:5:E)  I think it's definitely good as a second opinion. (3:1:B)  That would unsettle me [...] if something completely different comes out as a treatment suggestion or what I see in an MRI image or something like that, then it would make me very insecure and then I would want to make sure. Be it through the senior physician or that I can just have a look: How does this AI come up with this? And if that doesn't work, then it's unfavorable for the procedure. Then you have to rely on the senior consultant again and maybe the head physician [...] and then I'm back where I was before without AI. (2:1:3)  I think it's good. I think you still have to use it very critically. So you can't just say, yes, this is new and it's definitely great. Instead, you should critically scrutinize every app, device or AI that is new [...]. But I do believe that it can be a huge advantage for medicine. (3:1:A) |
|  | Data aspects | But the problem is also that many people are not even aware that all this data is stored and where it is stored. (3:1:1)  And things like data protection and so on are really legal and political problems. Inevitably, framework conditions will be created so that this can be fully utilized, I think. So there's no way around it. (2:2:5) |
|  | ELSI aspects^a^ | At some point it becomes unethical not to use such things. [...] That's actually the point. Why are we always so afraid that we're not important enough? At some point, the doctor is no longer the all-knowing person. (2:3:1)  Not everything that is technically possible should be utilized and I believe that a great deal of social discourse will be required in the coming years. Discourse within the medical profession too. [...] We can't refuse [AI] and we have to deal with it and will inevitably have to integrate it, whether we think it's good or bad. Personally, I think it has great potential if we use it wisely. But we mustn't leave the technology to the technology itself, because [...] if the programmes continue to write themselves - I have a bit of 1984 and that sort of thing in my head - and you have to be careful. (1:3:B1)  What if you don't know why the AI came to this decision? With Ada, you can see that. But that doesn't mean you can see it that way everywhere, right? I mean, why was this diagnosis made? If I can't understand it, what does that do to me as a doctor? (2:1:2) |

^a^ ELSI=Ethical, Legal and Social Implications

| **Main Category** | **Subcategories** | **Anchor Quotes (ID)** |
| --- | --- | --- |
| Overall Course Assessment | Learning Experience | It's often the case that you're told things and then you have to memorize them. And here it was more the case that you were given information but then had to think about it yourself, for example to discuss it or draw a picture or whatever. And that's a completely different kind of learning, which unfortunately we don't usually do that much of in our degree programs. So I thought it was really good. Because these are actually skills that you should have and not that you can somehow memorize a book. (2:1:3)  Actually, this shouldn't be an elective subject, but a compulsory subject. (1:3.B1)  So for me the greatest learning was through the discussions. (2:3:1) |
|  | Learning Success | I was also super satisfied with the week overall. I also learnt a lot of new things that I didn't know before. So just the knowledge and getting to know things that you didn't know at all, but that you hadn't really dealt with. Maybe you've heard about it before, but the fact that you were really actively involved was really good and I think I can now better judge what is perhaps useful and what is not. What do I need to look out for? Is it just this data protection thing or is it really useful? (1:3:B1)  I also thought the topic itself was a great advantage because I personally, and I think many others did too, hadn't really dealt with it before and have now actually realized within the five days what a big topic it is for us. And when I think now that there are other students who are just like us before the week, who had no insight at all into what we learnt, then I think that just addressing the topic and talking about it has a huge learning effect. (2:2:2)  I'm just glad that I had this week, because it really showed me what we don't learn at university. And how big the topic actually is for us. Of course, we don't have one hundred per cent solutions and I don't think any of us know now how we'll deal with it completely in five years' time, but I don't think that's the aim of it either, but simply that we've been sensitized to it a little so that we can now perhaps think about it during our specialist training or even in our final year, where can it go? And how do we deal with it personally? (2:2:2) |
|  | Structure | So I thought it was actually great in terms of time. Conversely, I would have liked to have stayed half an hour or an hour longer in some cases, just to talk to the people who are basically the experts again, because sometimes there wasn't enough time to ask questions. (2:3:2)  I thought everything was really well planned. Yes. Even with the rotations and stuff, I liked that. Because there was a lot of variety. (3:6:A) |
| Overall Course Assessment (Table continued) | Content | I generally found it very exciting that so many areas were covered. It was never really boring or anything, there was new input every day. (3:4:A)  I also thought it was good that a lot of external people were there, just to, I don't know, talk to those affected [patients], to talk to an app developer, to talk to the data protection officer, because it also brings in a lot of other perspectives. (2:1:1) |
|  | Methods | The nature of the discussion is simply different. Here you have time to talk to the experts, to talk about it. In a lecture, you sit there and are bombarded. Someone is talking at the front, not at your level, so to speak. And here it's just like that, very much at eye level. You can talk openly about things and, yes, it's more problem-orientated here. At university, it's simple: memorize the facts and then you pass the exam. And here it's just somehow – the quality is simply better. (2:2:5)  I'm very excited about the revised version of the e-book and I think it's a tool and an opportunity that could or should be used in many more areas. That there is also something like this in this form, where the most important information is summarized well in short, concise texts and sections. (1:3:B2)  What I also thought was great was this interactivity, that we could also try out this robot, so that was really cool. Or these [VR] headsets, where you could stand here 360 degrees in the operating theatre. I thought that was really cool, because I'd already heard about VR before, but the fact that you're actually standing in the operating theatre and the gall bladder is being operated on laparoscopically and you're allowed to get a taste of this DaVinci robot and do some dexterity exercises, I thought that was really impressive. (2:1:4) |
|  | Conclusion | I hope that the subject will be continued in this way, perhaps even expanded. That would be really great. If it can somehow reach all students and not just the lucky twelve who are then accepted into the compulsory elective subject. Because it's a great subject. [This professor] is as committed as you rarely experience. Your whole team. You're really into it and you all support it and are fully involved. And also that you come from such different backgrounds. I found that very enriching and great. (3:1:A) |
